# Impact factor and citation metrics in phase III cancer trials

**DOI:** 10.18632/oncotarget.28044

**Published:** 2021-08-31

**Authors:** Joseph Abi Jaoude, Ramez Kouzy, Michael Rooney, Petria Thompson, Roshal Patel, Maddie C. Turner, Marc Ghabach, C. David Fuller, Bruce D. Minsky, Cullen M. Taniguchi, Ethan B. Ludmir

**Affiliations:** ^1^The University of Texas MD Anderson Cancer Center, Houston, TX, USA; ^*^These authors contributed equally as first authors; ^#^These authors contributed equally as senior authors

**Keywords:** oncology, journal impact factor, clinical trials, FDA, industry

## Abstract

Purpose: Journal impact factor (IF) is often used to measure research quality and importance. We assessed trial factors associated with the publication of cancer trials in journals with higher IF and publications receiving higher citations.

Materials and Methods: Cancer-specific phase III RCTs were screened through https://clinicaltrials.gov. We identified trials with published primary endpoints, along with their corresponding journal IF and relative citation ratio (RCR).

Results: Seven-hundred ninety manuscripts were included in our study. Trials that met their primary endpoint were more commonly published in journals with higher IF (Median IF: positive trials 35.4 vs. negative trials 26.3, *P* < 0.001). Furthermore, trials that led to subsequent FDA drug approvals were also published in journals with higher IF (Median IF: 59.1 vs. 26.3 in trials not leading to FDA approvals, *P* < 0.001). When analyzing RCR, trial positivity (meeting primary endpoint) was not associated with increased citations on multivariable analysis (*P* = 0.56). Lastly, publications of trials leading to FDA approvals (*P* < 0.001), and publications of trials in journals with higher IF (*P* < 0.001) were associated with increased RCR.

Conclusions: Positive trials are commonly published in journals with high IF, but do not necessarily lead to increased citations. Moreover, trials published in journals with higher IF are more likely to receive increased citations.

## INTRODUCTION

Clinical trial reporting remains the cornerstone of disseminating clinical cancer research. The journal that ultimately publishes trial results plays a key role in the dissemination of scientific information. Generally, trialists and sponsoring bodies aim to publish trial results to the widest possible audience, which often involves aiming for publication in journals with a higher impact factor (IF) [1]. Journal IF is commonly perceived as a marker of research prestige and quality [2, 3]. Thus, the selection of manuscripts published in journals with high IF is based on a wide array of factors, including study impact, novelty, design, and quality. Furthermore, owing to the nature of IF being highly based on citations, many alternate factors could play a role in selecting which trial reports to publish in high-impact journals [4, 5]. Despite these considerations, data on IF and cancer trial reporting remain scarce. In this study, we investigated factors associated with the publication of trial results in journals with higher IF. We also sought to analyze trial factors associated with cancer trial publication leading to a higher relative citation ratio (RCR).

## RESULTS

### Journal impact factor

**Figure 1:**
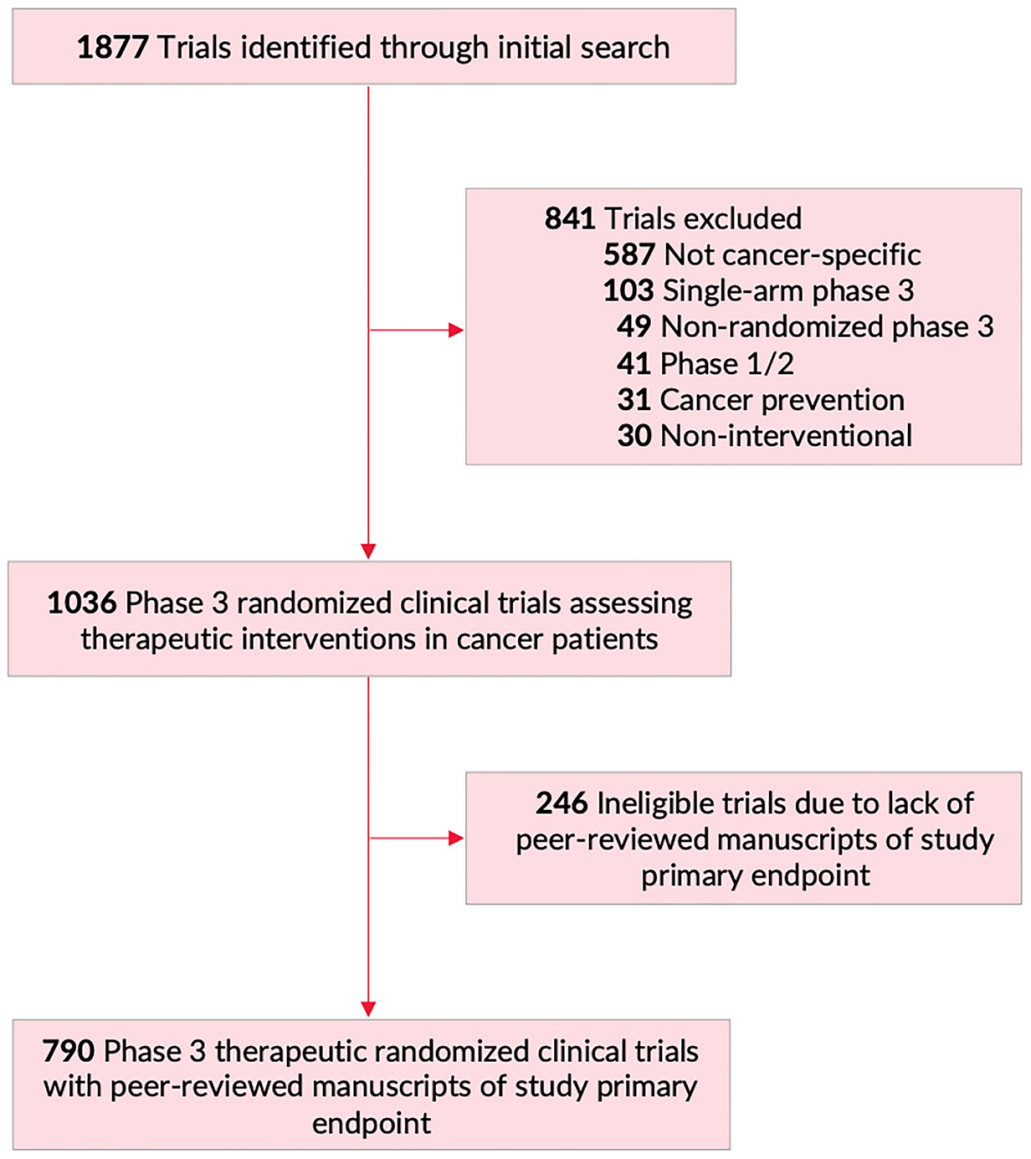
Flowchart of trial screening and inclusion. Of 1877 trials identified on https://clinicaltrials.gov/ (February 20, 2020), 841 were excluded, for a final total of 1036 phase III randomized clinical trials assessing therapeutic interventions in patients with cancer. Of those, 790 trials had peer-reviewed manuscripts of primary study endpoint, and were included in primary analysis for this manuscript.

In total, 790 manuscripts were included in our study (Figure 1). Table 1 presents trial-related factors associated with publication of trial results in journals with higher IF. On univariate analysis, trials receiving industry funding tended to publish in journals with higher IF compared to trials with no industry funding (Median: 26.3 for both, IQR: [14.2;59.1] vs. [6.1;35.4], respectively, *P* < 0.001). Trials of genitourinary cancers were published in journals with higher IF compared to other disease sites (Median: 35.4, IQR: [22.4;70.7], *P* = 0.01; Table 1). When analyzing intervention modality, trials studying supportive care interventions (Median: 6.2, IQR: [2.8;26.3], *P* < 0.001) tend to publish in lower-IF journals compared systemic therapy trials (Median: 26.3, IQR: [22.4;59.1], *P* < 0.001). Trials that met their primary endpoints (Median: 35.4, IQR: [26.3;70.7], *P* < 0.001), and trials leading to subsequent FDA drug approvals (Median: 59.1, IQR: [35.4;70.7], *P* < 0.001) were more commonly published in journals with higher IF. On multivariable regression modeling, cooperative-group sponsorship, genitourinary cancer trials, trial positivity (meeting the primary endpoint), and trials leading to subsequent FDA approval were all independently associated with publication of trial results in higher IF journals (Table 1).

**Table 1:** Trial factors associated with phase III cancer trials publishing their primary endpoint results in journals with higher impact factor

Trial Variables	JIF (median [IQR])	*P*	Multivariable Regression (β slope [95% CI])	*P*
**Industry-funding^a^**				
No (*n* = 184)	26.3 [6.1;35.4]	<0.001	–	
Yes (*n* = 606)	26.3 [14.2;59.1]		0.8 [−3.5;5.2]	0.71
**Cooperative-support^a^**				
No (*n* = 555)	26.3 [14.2;59.1]	0.33	–	
Yes (*n* = 235)	26.3 [12.1;51.2]		9.3 [5.4;13.1]	<0.001
**Disease Site**				
Breast (*n* = 147)	26.3 [8.9;35.4]	0.56	1.1 [−3.2;5.4]	0.62
Gastrointestinal (*n* = 98)	26.3 [14.2;35.4]	0.97	2.8 [−2.1;7.7]	0.27
Genitourinary (*n* = 95)	35.4 [22.4;70.7]	0.01	8.1 [3.2;13.0]	0.01
Head and Neck (*n* = 28)	26.3 [11.5;35.4]	0.67	2.7 [−4.7;10.1]	0.47
Hematologic (*n* = 155)	26.3 [16.6;59.1]	0.32	1.0 [−3.2;5.3]	0.63
Lungs (*n* = 114)	26.3 [14.2;35.4]	0.61	2.0 [−2.8;6.8]	0.41
**Modality^b^**				
Systemic Therapy^c^ (*n* = 621)	26.3 [22.4;59.1]	<0.001	2.7 [−22.8;28.2]	0.83
Radiation Therapy (*n* = 23)	26.3 [22.4;35.4]	0.76	4.0 [−22.5;30.5]	0.77
Surgery (*n* = 8)	10.9 [5.1;47.3]	0.33	−3.0 [−31.8;25.9]	0.84
Supportive Care^d^ (*n* = 136)	6.2 [2.8;26.3]	<0.001	−11.5 [−37.1;14.1]	0.38
**Trial Results**				
Negative (*n* = 357)	26.3 [6.2;26.3]	<0.001	–	
Positive (*n* = 411)	35.4 [26.3;70.7]		7.8 [4.6;11.0]	<0.001
**FDA Approval**				
No (*n* = 565)	26.3 [6.2;35.4]	<0.001	–	
Yes (*n* = 225)	59.1 [35.4;70.7]		22.4 [18.7;26.0]	<0.001

Abbreviations: JIF: journal impact factor; IQR: interquartile range; FDA: Food and Drug Administration. ^a^Industry funding and cooperative group sponsorship were considered independent variables as certain trials were both industry-funded and performed through a multi-institutional cooperative group. ^b^Modality addressed the primary intervention as part of the randomization. ^c^Systemic therapy trials, including chemotherapy, targeted systemic agents, immunotherapy, and others, accounted for most trials by modality; they used systemic therapies to improve disease-related outcomes (e.g., overall survival, disease-free survival). ^d^Supportive care trials were those where the intervention aimed to reduce disease- or treatment-related toxic effects as the primary endpoint.

### Relative citation ratio

We further sought to examine similar associations between trial-related factors and citation rates as reported through the RCR. Univariate analysis demonstrated that publications of industry-sponsored trials had higher average RCR (Median RCR: 8.8 vs. 3.3 for non-industry-funded trials, *P* < 0.001). On the other hand, cooperative-group sponsorship was associated with lower RCR (Median RCR: 4.6 vs. 8.4 in non-cooperative-sponsored trials, *P* < 0.001). By disease site, trials assessing patients with gastrointestinal malignancies (Median RCR: 11.0, IQR: [3.8;24.5], *P* = 0.01) led to publications receiving higher RCR, while breast cancer trials tended to have lower RCR (Median RCR: 4.7, IQR: [2.3;12.8], *P* = 0.01). Similar to the IF analysis, supportive care trials also tended to have lower RCR compared to systemic therapy trials (Median RCR: 2.6 vs. 9.1, respectively, *P* < 0.001 for both). Furthermore, trial positivity and FDA drug approval were associated with publication with higher RCR (*P* < 0.001 for both). Multivariable analysis confirmed that disease site (cancers other than breast and hematological), subsequent FDA drug approval, and increased journal IF were independently associated with publications leading to higher RCR (Table 2). Interestingly, trial positivity was not independently associated with RCR on multivariable analysis (*P* = 0.56).

**Table 2:** Trial factors associated with phase III cancer trial publications receiving higher relative citation ratio

Trial Variables	RCR (median [IQR])	*P*	Multivariable Regression (β slope [95% CI])	*P*
**Industry-funding^a^**				
No (*n* = 184)	3.3 [1.6;8.3]	<0.001	–	
Yes (*n* = 606)	8.8 [3.1;25.0]		0.49 [−4.7;5.7]	0.85
**Cooperative-support^a^**				
No (*n* = 555)	8.4 [2.8;24.6]	<0.001	–	
Yes (*n* = 235)	4.6 [2.2;13.7]		−3.7 [−8.4;0.9]	0.11
**Disease Site**				
Breast (*n* = 147)	4.7 [2.3;12.8]	0.01	−10.2 [−15.3;−5.0]	<0.001
Gastrointestinal (*n* = 98)	11.0 [3.8;24.5]	0.01	−0.6 [−6.4;5.3]	0.85
Genitourinary (*n* = 95)	8.2 [3.4;37.6]	0.03	−3.6 [−9.5;2.3]	0.23
Head and Neck (*n* = 28)	4.2 [1.9;20.0]	0.36	−5.8 [−14.7;3.1]	0.20
Hematologic (*n* = 155)	7.9 [2.6;18.9]	0.96	−13.0 [−18.1;−7.9]	<0.001
Lungs (*n* = 114)	7.5 [2.6;20.5]	0.51	3.5 [−2.2;9.3]	0.23
**Modality^b^**				
Systemic Therapy^c^ (*n* = 621)	9.1 [3.2;25.2]	<0.001	2.3 [−40.3;44.8]	0.92
Radiation Therapy (*n* = 23)	8.6 [4.4;20.0]	0.58	3.5 [−40.0;47.0]	0.88
Surgery (*n* = 8)	4.9 [1.4;34.1]	0.86	14.6 [−31.0;60.3]	0.53
Supportive Care^d^ (*n* = 136)	2.6 [1.1;5.1]	<0.001	2.6 [−30.9;45.2]	0.90
**Trial Results**				
Negative (*n* = 357)	4.0 [1.8;8.6]	<0.001	–	
Positive (*n* = 411)	14.4 [4.1;34.3]		1.2 [−2.7;5.1]	0.56
**FDA Approval**				
No (*n* = 565)	4.1 [1.9;9.3]	<0.001	–	
Yes (*n* = 225)	28.8 [14.3;49.3]		17.5 [12.7;22.3]	<0.001
**JIF**	R = 0.60XXXR^2^ = 0.37	<0.001	0.6 [0.5;0.7]	<0.001

Abbreviations: RCR: relative citation ratio; IQR: interquartile range; FDA: Food and Drug Administration. ^a^Industry funding and cooperative group sponsorship were considered independent variables as certain trials were both industry-funded and performed through a multi-institutional cooperative group. ^b^Modality addressed the primary intervention as part of the randomization. ^c^Systemic therapy trials, including chemotherapy, targeted systemic agents, immunotherapy, and others, accounted for most trials by modality; they used systemic therapies to improve disease-related outcomes (e.g., overall survival, disease-free survival). ^d^Supportive care trials were those where the intervention aimed to reduce disease- or treatment-related toxic effects as the primary endpoint.

## DISCUSSION

The IF, originally proposed in *Science* in 1955, represents an index measured based on the average number of citations per article published over a specific period of time [4, 5]. Despite the development of different tools to assess the quality of articles published in a journal, the IF remains among the most important and commonly-utilized journal bibliometrics [6–8]. Our data show that cancer trials assessing systemic therapy, trials that meet their primary endpoint, and trials leading to FDA drug approvals tend to publish in journals with higher IF. Along the same lines, these data demonstrate that publications of trials leading to FDA approvals and trial publications in journals with higher IF tend to have a higher RCR.

A journal’s IF is frequently associated with journal and research prestige, and is considered to correlate with research quality [2, 3]. Furthermore, readers are often subject to the so called *“prestigious journal bias”* and overestimate results posted in journals with high IF [9]. Our data show that trials assessing systemic therapy tend to publish their results in journals with substantially higher IF when compared to trials assessing supportive care interventions (26.3 vs. 6.2, respectively). Through disproportionate publication of systemic therapy trials, higher-impact journals could be missing opportunities to highlight valuable results from different oncologic disciplines and interventions, most notably supportive care studies.

On univariate analysis, industry-funding was associated with results publishing in journals with higher IF. However, when we adjusted for multiple trial-factors, this association did not maintain statistical significance. This could be explained by the fact that a large proportion of industry-funded trials assess systemic therapy interventions and lead to FDA approvals, and those factors (systemic therapy modality, subsequent FDA approval) could be driving the association between industry sponsorship and IF. Furthermore, cooperative-group-supported trials published results in journals with higher IF. This pattern may reflect rigorous scientific merit of cooperative group studies or possibly an editorial bias toward publishing such trials in higher IF journals, among other potential explanations. Additionally, cooperative group sponsorship was associated with lower RCR, but this association was not maintained on multivariable analysis.

Our results demonstrate that trials meeting their primary endpoints and trials leading to subsequent FDA drug approvals commonly publish their results in higher-IF journals. This could be related to the high volume of manuscript submissions to higher-impact journals, which would drive more exclusive publication of trials that are practice-changing, and have a direct impact on patient management. Nevertheless, this could still shift attention away from negative trials, despite negative results retaining high value and importance to the advancement of cancer care [10]. Reinforcing clinical practice through negative data from prospective trials is an integral part of the scientific enterprise [11]. In an effort to mitigate publication bias of positive trials, journals could consider offering trialists the choice of guaranteed publication of results as long as the trial follows a pre-determined and sound methodology to completion, irrespective of final results. As such, trial publication would be dependent on factors such as accrual, toxicity monitoring, and proper statistical analysis, rather than on the results obtained. This would give positive and negative trials equal chances at highlighting their results, while advocating for study design as a key priority for subsequent publication in a high-visibility/high-impact journal.

Interestingly, despite trial positivity being associated with higher-IF publication, we show that after adjusting for multiple trial factors, trial positivity was not associated with increased RCR. By contrast, FDA approval was associated with both increased IF and RCR. This discrepancy could be explained by the possibility that positive trials that do not lead to FDA approvals often examine already-approved drugs, and as such may be studying fewer novel interventions. This may lead to manuscripts having fewer subsequent citations, despite having positive trial results. Moreover, our analysis shows that publications in journals with higher IF receive higher citations. Nevertheless, some inconsistencies in IF and citations are expected due to the differences in disease prevalence and number of active investigators in a particular research niche. In that regard, understanding the intricacies leading to publications of cancer trials in high-impact journals is crucial.

While the journal IF is a broadly-useful scientific bibliometric, a potential issue with IF is in the nature of its calculation, which is solely based on subsequent article citations [12]. As such, a journal IF could be influenced by multiple alternate factors other than research quality. Modern adjustments to the journal IF metric, with a higher emphasis on research quality and design could be considered to alleviate the influence of external variables on manuscript publication. Moreover, separate bibliometric measures could be used in different medical disciplines to allow proper comparisons of research within different fields.

A primary limitation of this study is that journal IF is not static, and subject to change (including inflation) over time. As such, some journals might have had different IF at the time of publication than as analyzed in this report. Moreover, certain trial factors that were not included in our model may have affected our results in ways that we could not adjust for. Finally, our data are limited by the fundamental heterogeneity of the publication process of individual trials, which involves exceptionally complex interactions between multiple parties. Nevertheless, our study remains the largest to analyze factors associated with phase III cancer trial publications in high-impact journals.

In conclusion, trials meeting their primary endpoints and trials leading to subsequent FDA drug approvals are often published in journals with a higher IF. Analyzing those factors associated with differential publication of oncologic trial results may help in understanding the complex role of bibliometrics in publishing cancer trials.

## MATERIALS AND METHODS

### Study design

We performed a database query through the https://clinicaltrials.gov registry to search for oncologic phase 3 randomized clinical trials (RCTs) on February 20, 2020. The following advanced search parameters were used: other terms: “cancer”; study type: “All Studies”; status: excluded “Not yet recruiting”; phase: phase 3; Study results: “With Results.” This query yielded a total of 1,877 trials (Figure 1). We screened all trials for therapeutic, cancer-specific, phase 3, randomized, multi-arm trials. Trial features were assessed through data from https://clinicaltrials.gov, the trial’s protocol, and/or the primary publication of trial results, when available [13]. After screening, 1,036 trials met inclusion criteria for this study, and of these 790 trials had a published manuscript reporting on the trial’s primary endpoint results (Figure 1). For every manuscript published, we identified the corresponding journal and its impact factor, along with the corresponding RCR using data collected from Clarivate and the National Institutes of Health’s iCite website [14]. No institutional review board approval was required from our home institution (the University of Texas MD Anderson Cancer Center Institutional Review Board). All data were publicly available; no patient health information was obtained or utilized, and therefore no informed consent was required.

### Statistical analysis

The Mann-Whitney *U*-tests were used to assess the association between categorical trial-related factors and journal IF as well as RCR. Linear regression was used to assess the association between journal IF and RCR. Lastly, multivariable linear regression modeling was performed to identify factors independently associated with journal IF as well as increased RCR. Statistical significance was set *a priori* at a two-sided *α* = 0.05. All analyses were performed using IBM SPSS version 26.0 [15].

## Abbreviations

FDA: food and drug administration
IF: Impact Factor
IQR: interquartile range
RCT: randomized clinical trial
RCR: relative citation ratio
